# Varicella–Zoster Virus Infection in Pregnancy: Maternal, Fetal, and Neonatal Implications in the Vaccination Era

**DOI:** 10.3390/microorganisms14081745

**Published:** 2026-08-08

**Authors:** Isadora Rodrigues Almeida, Camila Silva Belo, Tammy Caram Sabatine, Thamy Cristina Campos, Annie Stefanelli, Liris Naomi Noguchi, Giuliana Augustinelli Sales, Gustavo Yano Callado, Angélica Lemos Debs Diniz, Roberta Granese, Edward Araujo Júnior, Antonio Braga

**Affiliations:** 1 Discipline of Woman Health, Municipal University of São Caetano do Sul (USCS), São Caetano do Sul 09521-160, SP, Brazil; isadealrodrigues@gmail.com (I.R.A.); camilasilvabelo@gmail.com (C.S.B.); tammycaramsabatine@hotmail.com (T.C.S.); thamycristinacampos@yahoo.com.br (T.C.C.); anniestefanelli@outlook.com (A.S.); giu.sales@hotmail.com (G.A.S.); araujojred@terra.com.br (E.A.J.); 2 Discipline of Gynecology and Obstetrics, Santo Amaro University (UNISA), São Paulo 04829-300, SP, Brazil; lirisnaomin@gmail.com; 3 Department of Medicine, Faculty of Medicine, Albert Einstein Israelite College of Health Sciences (FICSAE), Albert Einstein Israelite Hospital, São Paulo 05652-900, SP, Brazil; gycallado@gmail.com; 4 Department of Obstetrics and Gynecology, Federal University of Uberlândia (UFU), Uberlândia 38405-320, MG, Brazil; angelicadiniz@ufu.br; 5 Department of Biomedical and Dental Sciences and Morphofunctional Imaging, “G. Martino” University Hospital, 98100 Messina, Italy; roberta.granese@unime.it; 6 Department of Obstetrics, Paulista School of Medicine, Federal University of São Paulo (EPM-UNIFESP), São Paulo 04023-062, SP, Brazil; 7 Department of Gynecology and Obstetrics, School of Medicine, Federal University of Rio de Janeiro (UFRJ), Rio de Janeiro 22240-003, RJ, Brazil; 8 Department of General and Specialized Surgery, School of Medicine and Surgery, Federal University of the State of Rio de Janeiro (UNIRIO), Rio de Janeiro 22290-240, RJ, Brazil; 9 Postgraduate Program in Applied Health Sciences, University of Vassouras (Univassouras), Vassouras 27700-000, RJ, Brazil

**Keywords:** varicella–zoster virus, chickenpox, pregnancy, congenital varicella syndrome, neonatal varicella, acyclovir, post-exposure prophylaxis

## Abstract

Infection by the varicella–zoster virus (VZV) during pregnancy is uncommon but clinically relevant, since it may compromise both the mother and the fetus. Although varicella is generally benign and self-limited in childhood, the physiological and immunological changes in pregnancy predispose individuals to more severe forms of the disease, with pneumonia representing the main maternal complication and a leading cause of morbidity and mortality. Vertical transmission may result in congenital varicella syndrome, the most severe fetal consequence, whose risk is greatest between the 13th and 20th weeks of gestation, or in severe neonatal varicella when maternal infection occurs in the peripartum period. This article presents a narrative review of the literature. To enhance methodological transparency and reproducibility, key principles of the Preferred Reporting Items for Systematic Reviews and Meta-Analyses (PRISMA) were applied to the literature search and study selection process. Searches were conducted in the PubMed/MEDLINE and SciELO databases, and 32 studies were included in the narrative synthesis. The review addresses the virology, pathophysiology, and epidemiology of VZV infection, including differences between temperate and tropical regions, as well as its clinical manifestations and maternal, fetal, and neonatal complications. Diagnosis is predominantly clinical, with the polymerase chain reaction being the most sensitive and specific confirmatory method and serology being useful mainly for the assessment of maternal immunity. Management encompasses the assessment of susceptibility, post-exposure prophylaxis according to current guideline recommendations, and early antiviral treatment of established infection. Preconception and postpartum vaccination remain the main preventive strategies. Despite recent advances, important gaps persist regarding prophylactic strategies and the long-term safety of antivirals during pregnancy.

## 1. Introduction

The Varicella–Zoster virus (VZV) is a human double-stranded DNA α-herpesvirus responsible for two distinct clinical presentations: varicella (chickenpox), a primary infection that is predominantly pediatric, and herpes zoster, which results from viral reactivation after latency in the sensory ganglia [1,2]. Although benign and self-limited in childhood, varicella acquires significant risk characteristics when it occurs during pregnancy, since it can compromise both the mother and the fetus [1,3].

Varicella–zoster virus (VZV) infection during pregnancy is associated with clinically important maternal, fetal, and neonatal morbidity, with the spectrum and severity of complications largely determined by the gestational age at infection and the degree of passive maternal antibody transfer. Although most infections are self-limited, severe outcomes may occur in both the mother and the offspring, underscoring the importance of timely prevention, diagnosis, and management [1,2,3].

In this context, early and accurate diagnosis is fundamental, since late recognition of the disease may delay therapeutic and prophylactic measures capable of preventing adverse outcomes [4]. The present review aims to provide a comprehensive overview of varicella–zoster virus infection during pregnancy, addressing its virology, epidemiology, pathophysiology, maternal, fetal, and neonatal implications, diagnostic methods, prevention strategies, and current clinical management, with the aim of supporting evidence-based decision-making in obstetric practice.

## 2. Methods

This article is a narrative review of the literature designed to provide a comprehensive and clinically oriented overview of varicella–zoster virus (VZV) infection during pregnancy. To enhance the transparency and reproducibility of the review process, a structured literature search was performed. However, the review was not conducted as a systematic review, and therefore it did not follow the full methodological requirements of the Preferred Reporting Items for Systematic Reviews and Meta-Analyses (PRISMA) statement, nor was a formal PRISMA checklist or flow diagram applied.

A structured search of the PubMed/MEDLINE and SciELO databases was conducted through March 2026 using combinations of Medical Subject Headings (MeSH) and free-text terms. The search strategy combined terms related to varicella–zoster virus (“Varicella-Zoster Virus” OR varicella OR chickenpox) with terms related to pregnancy and congenital infection (pregnancy OR pregnant OR “congenital varicella syndrome” OR “neonatal varicella”), and prevention and treatment (“post-exposure prophylaxis” OR VariZIG OR VZIG OR acyclovir OR valacyclovir), using the Boolean operators AND and OR as appropriate. Additional relevant publications were identified through manual searches of the reference lists of selected articles and through international clinical guidelines and documents issued by international scientific societies and public health agencies. The search was limited to studies published in English, Portuguese, or Spanish, involving human subjects.

Evidence sources included international clinical guidelines, observational studies, systematic reviews and meta-analyses, narrative reviews, and selected case reports considered informative for rare clinical presentations or management challenges. Priority was given to publications with clear methodological descriptions, direct relevance to the objectives of this review, applicability to clinical practice, and consistency with recommendations from major international scientific societies whenever available.

The retrieved literature was synthesized narratively, integrating evidence from the selected publications with recommendations from major international scientific societies to provide an updated and clinically applicable overview of maternal, fetal, and neonatal VZV infection. Because this was a narrative review, no formal assessment of methodological quality or certainty of evidence was undertaken.

As this is a literature review based exclusively on secondary data in the public domain, the study did not require submission to a research ethics committee.

## 3. Virology and Pathophysiology of the Varicella–Zoster Virus

VZV is a highly contagious DNA herpesvirus that presents specific virological and pathophysiological characteristics during pregnancy, with important implications for the mother and fetus [5].

### 3.1. Virological Characteristics

VZV is an enveloped double-stranded DNA herpesvirus belonging to the family *Herpesviridae*, subfamily *Alphaherpesvirinae* [6]. As a member of this family, the virus has the capacity to establish latency after primary infection and subsequently reactivate. The enveloped viral structure allows the virus to be relatively labile in the external environment but highly efficient in person-to-person transmission [5].

The virus has tropism for epithelial cells of the respiratory tract during primary infection and, subsequently, for neurons of the dorsal and cranial sensory ganglia, where it establishes latency [5,7]. Viral replication occurs initially in the respiratory mucosa, followed by a primary viremia that disseminates the virus to the reticuloendothelial system, and subsequently a secondary viremia that results in the specific manifestation of varicella [1,4].

### 3.2. Mechanisms of Transmission

VZV is transmitted efficiently, with a transmission rate of 60–90% among susceptible (seronegative) contacts. Transmission occurs mainly through respiratory droplets and through direct contact with vesicular lesions. The incubation period after exposure is 10–20 days, with a mean of 14 days. The infectious period begins 48 h before the appearance of the exanthem and persists until all vesicles have crusted over [5].

Vertical transmission can occur during maternal primary infection (varicella), when the virus crosses the placenta during maternal viremia, in the incubation period, and during the exanthem. The vertical transmission rate varies according to gestational age: studies demonstrate detection of IgM in 5%, 10%, and 25% of newborns after maternal varicella in the first, second, and third trimesters, respectively [8].

In studies using PCR in amniotic fluid, the transmission rate was 8.4% in the first 24 weeks of gestation [8]. Transmission of maternal herpes zoster is extremely rare, since it requires contact with an open lesion and the viral load is significantly lower in reactivation compared with primary infection [5]. In 380 prospective cases of herpes zoster in pregnancy, there were no cases of congenital varicella syndrome (CVS) or herpes zoster in newborns [8].

**Figure 1:**
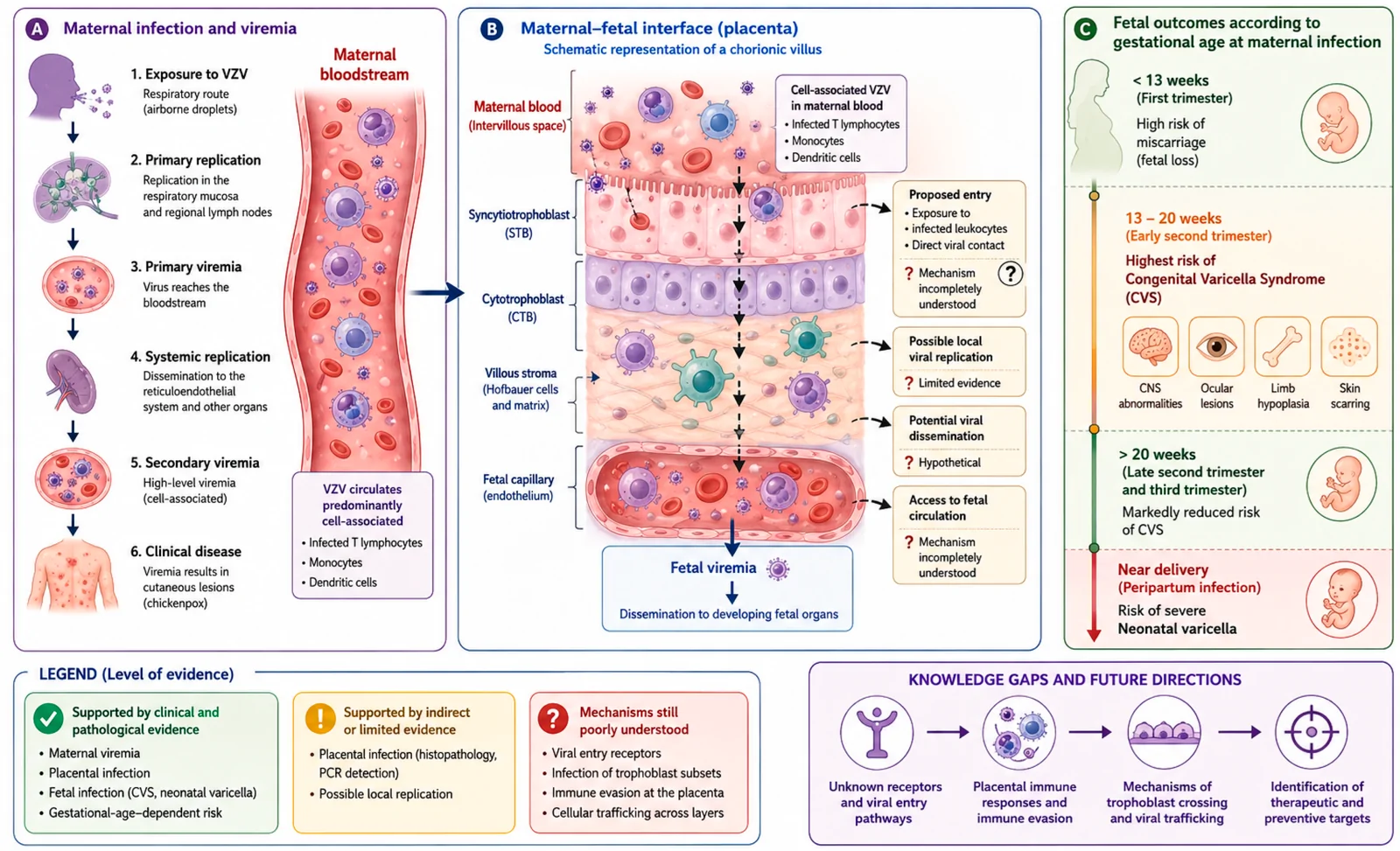
Current understanding of the proposed mechanisms of transplacental transmission of varicella–zoster virus (VZV) during pregnancy. The figure summarizes the current evidence regarding maternal VZV infection, placental transmission, and fetal outcomes according to gestational age at maternal infection. (**A**) Following respiratory acquisition, VZV undergoes primary replication in regional lymphoid tissue, leading to primary and secondary cell-associated viremia that disseminates the virus systemically. (**B**) At the maternal–fetal interface, maternal viremia is considered a prerequisite for placental infection. Current evidence suggests that infected maternal leukocytes may facilitate viral exposure to the syncytiotrophoblast, with subsequent dissemination across placental tissues to the fetal circulation. However, the precise cellular and molecular mechanisms governing viral entry, trophoblast infection, immune evasion, and transplacental passage remain incompletely understood. (**C**) The risk and clinical consequences of fetal infection vary according to gestational age, ranging from miscarriage in early pregnancy to congenital varicella syndrome between 13 and 20 weeks of gestation, with a markedly reduced risk thereafter, whereas maternal infection near delivery is primarily associated with severe neonatal varicella. The bottom panels distinguish mechanisms supported by clinical and pathological evidence from those supported by indirect evidence and highlight the principal knowledge gaps that warrant future investigation. Abbreviations: CTB, cytotrophoblast; CVS, congenital varicella syndrome; PCR, polymerase chain reaction; STB, syncytiotrophoblast; VZV, varicella–zoster virus.

Although maternal viremia is recognized as the prerequisite for transplacental transmission, the precise cellular and molecular mechanisms by which VZV crosses the placental barrier remain incompletely understood. Current evidence suggests that viral dissemination across the maternal–fetal interface likely involves sequential interactions between maternal immune cells, trophoblast layers, and placental tissues, although several key steps remain hypothetical (Figure 1).

### 3.3. Viral Latency and Reactivation

After primary infection (varicella), VZV remains latent in the dorsal and cranial sensory ganglia for life [5,7]. During latency, the viral genome persists in the neurons in an episomal form, with minimal or absent viral gene expression [7].

Reactivation of VZV results in herpes zoster (shingles), which occurs in approximately 30% of people who have had varicella over the course of their lives [7]. Reactivation manifests as an erythematous vesicular exanthem in a specific dermatomal distribution [5]. Herpes zoster is less contagious than varicella, but the VZV from the lesions can be transmitted by direct contact or through the air and cause varicella in susceptible contacts [7].

Recent evidence suggests that asymptomatic reactivation of VZV occurs frequently during the third trimester of pregnancy. In a study that analyzed the saliva of pregnant women, no viral DNA was detected in the first trimester, but VZV DNA was identified in most of the samples (11/12 by nested PCR; 7/10 by qPCR) during the third trimester [9]. These features, which remain below the clinical thresholds in most patients, may provide an endogenous boost to immunity and, in this way, be beneficial [9].

### 3.4. Immunological Adaptations During Pregnancy

Pregnancy is associated with significant changes in maternal immunological and hormonal status [3]. Although the literature does not extensively detail the specific immunological mechanisms related to VZV in pregnancy, some clinical observations reflect these adaptations:

• Greater severity of maternal disease: Adults, including pregnant women, present more severe morbidity than children. Approximately 10–20% of pregnant women with varicella develop pneumonia, which represents a significant risk factor for maternal mortality, estimated at up to 40% [5]. This greater severity may reflect changes in cell-mediated immunity during pregnancy.
• Preserved humoral immunity: The antibody response to VZV develops within a few days after the onset of infection, and prior infection confers lifelong immunity against primary infection [5]. The transplacental transfer of maternal IgG antibodies is crucial for neonatal protection [5].
• Neonatal vulnerability: Neonatal VZV infection is associated with a high mortality rate when maternal disease develops from 5 days before delivery to 48 h postpartum, due to the relative immaturity of the neonatal immune system and the lack of protective maternal antibodies [5]. Two-thirds of newborns exposed in this period are infected, and half develop symptoms, including ulcerated, necrotic, or hemorrhagic lesions and/or systemic diseases (pneumonia, hepatic failure, encephalitis, and coagulopathy), with a historical mortality of approximately 30% before antiviral therapy [7].
• Viral reactivation: The frequent asymptomatic reactivation in the third trimester suggests that the immunological changes in pregnancy may favor viral reactivation, although it generally remains subclinical [9]. These features may represent a mechanism for the maintenance of maternal immunity. The incidence of varicella in pregnancy is relatively low (0.4–0.7 per 1000 pregnant women before universal vaccination) due to the high prevalence of natural immunity, and it is even lower currently with routine vaccination [5].

## 4. Epidemiology of Varicella in Pregnancy

### 4.1. Global Epidemiology

Varicella is a highly contagious viral infection caused by the VZV, classically occurring in childhood. Before the widespread introduction of vaccination, approximately 90% of cases occurred before 15 years of age, especially in countries with a temperate climate [4,10].

The seroprevalence of anti-VZV antibodies in adults exceeds 90% in most of Europe [4,11]. Among women of reproductive age, French studies demonstrated a seroprevalence between 95–98% [4], while studies in Spain and France reported immunity of 96.1% and 98.8%, respectively [2,12].

Despite frequent exposure to VZV during pregnancy, maternal primary infection remains relatively rare. The estimated incidence varies between 0.5–1.2 cases per 1000 pregnancies [2] and may reach 2–3 cases per 1000 pregnancies in the United Kingdom [10,11,12]. In the United States, in the pre-vaccine era, estimates vary between 1.6–4.6 cases per 1000 pregnancies [10,11,12].

Although only about 2% of varicella cases occur in adults, these individuals account for approximately 25% of VZV-related deaths [13]. Infection in pregnant women is associated with greater maternal morbidity, particularly due to varicella pneumonia, in addition to fetal and neonatal complications [4,13].

CVS is a rare but serious complication. A systematic review of 13 cohort studies estimated a global incidence of 0.59%, increasing to 0.84% when infection occurs in the first 20 gestational weeks [14]. Other studies describe an approximate risk of 2% for maternal infection between 0–20 weeks [4].

Maternal infection close to delivery represents an important neonatal risk factor. When the maternal exanthem occurs between five days before and two days after delivery, the newborn presents a high risk of severe neonatal varicella [4,13]. Before the routine introduction of anti-VZV immunoglobulin, neonatal mortality could reach 30%, decreasing to less than 7% after its use [13].

### 4.2. Impact of Vaccination Programs

Vaccination against varicella represents the main strategy for prevention of the disease [4]. The implementation of universal childhood vaccination programs has significantly modified the epidemiology of VZV, promoting a reduction in incidence, hospitalizations, and mortality [15].

In addition to individual protection, vaccination promotes herd immunity, reducing community viral circulation and decreasing the exposure of susceptible pregnant women [10].

However, insufficient vaccination coverage may cause a shift in the infection to older age groups, proportionally increasing the number of susceptible adults [15]. In Italy, vaccination coverage below 80% was associated with the maintenance of viral circulation and a possible expansion of the contingent of susceptible adults [15].

Italian data demonstrated national vaccination coverage of approximately 30.7% in 2015 and 46.06% in 2017. In parallel, studies identified seronegativity rates among pregnant women varying between 10–12%, higher than those observed in other European countries [15].

In the United States and the United Kingdom, a proportional increase in varicella morbidity in young adults was observed during the 1990s [10,11]. One of the proposed hypotheses is the increased immigration of susceptible individuals coming from tropical regions [11,12].

Universal childhood vaccination has fundamentally altered the force of infection of VZV by markedly reducing community viral circulation. As a consequence, susceptible individuals experience fewer natural exposures throughout childhood, resulting in substantial declines in disease incidence, hospitalizations, and mortality in countries with sustained high vaccine coverage. However, mathematical models and post-implementation surveillance have shown that when vaccination coverage is insufficient to interrupt transmission, a proportion of susceptible individuals may escape infection during childhood and remain susceptible into adolescence and adulthood. Because primary varicella infection is generally more severe in adults than in children, maintaining high vaccination coverage is essential to avoid this epidemiological age shift.

These epidemiological changes are particularly relevant in obstetric practice, as they directly influence the susceptibility profile of women of reproductive age. While countries with long-standing universal vaccination programs have observed marked reductions in maternal varicella, regions with heterogeneous vaccine uptake or large migrant populations from tropical areas may continue to harbor susceptible pregnant women, reinforcing the importance of preconception immunity assessment and postpartum vaccination.

The vaccination era has also introduced new clinical considerations. Breakthrough varicella may occur in previously vaccinated individuals but is generally characterized by milder clinical manifestations, lower viral burden, and a substantially lower risk of severe maternal and fetal complications than primary infection in susceptible women. Although vaccine-induced immunity differs immunologically from naturally acquired immunity, both provide effective protection against severe disease. Nevertheless, incomplete vaccine uptake and missed opportunities for preconception screening and postpartum immunization continue to maintain a small but clinically important population of susceptible women of reproductive age, highlighting the need for preventive strategies that extend beyond childhood vaccination programs.

### 4.3. Epidemiological Differences Between Temperate and Tropical Regions

The epidemiology of varicella differs substantially between temperate and tropical regions [10,11]. In temperate regions, infection occurs predominantly in childhood, with typical seasonality in winter and early spring [10,11]. In these countries, more than 90% of the population acquires immunity before 15 years of age [10]. On the other hand, in tropical and subtropical regions, primary infection tends to occur at older ages, including adolescence and adulthood [15,16]. Studies demonstrate a significantly lower seroprevalence in these locations, varying between 25–85% [10].

In India, it is estimated that more than 30% of the population over 15 years of age remains susceptible to VZV [16]. Serological studies identified seronegativity rates varying from less than 5% in the United States to 14–19% in Saudi Arabia, 26% in India, and approximately 50% in Sri Lanka [16]. These epidemiological differences result in a greater proportion of women of childbearing age susceptible to primary VZV infection in tropical countries [10,16]. In addition, migrant women coming from tropical regions present lower seroprevalence even when residing in temperate countries [4,11].

An Irish study involving 7980 women demonstrated a global seroprevalence of 93%, but only 80% among women originating from Sub-Saharan Africa, Asia, and Central and Eastern Europe [4]. The main epidemiological differences between temperate and tropical/subtropical regions are summarized in Table 1.

### 4.4. Risk Factors for Maternal Infection

The main risk factor for primary VZV infection during pregnancy is the absence of prior immunity [4]. Among the factors associated with maternal susceptibility, the following stand out: origin in tropical regions, immigration, low population vaccination coverage, and close contact with infected individuals [11,12,16].

Transmission occurs mainly in household and occupational environments with frequent contact with children [2]. Health professionals and women with young children present a greater risk of exposure. Conditions of crowding, travel to endemic areas, and living with infected individuals also increase the risk of transmission [2].

Varicella pneumonia represents the main severe maternal complication. It occurs in up to 20% of infected pregnant women [13]. Pregnant women who smoke and women with more than 100 skin lesions present a greater risk for this complication [13].

In addition, the mortality associated with VZV pneumonia can reach approximately 14% even with treatment [13]. Other authors report that pregnant women present a five-fold greater risk of a fatal outcome compared with non-pregnant women [17].

States of immunosuppression, including HIV infection, use of immunosuppressants, and chemotherapy, increase both the risk of primary infection and that of viral reactivation [2]. Other factors related to VZV reactivation include advanced maternal age, chronic diseases, physical or emotional stress, and COVID-19 infection [2]. The main risk factors and their reported association are summarized in Table 2.

## 5. Clinical Manifestations of Varicella in Pregnancy

### 5.1. Maternal Primary Varicella Infection

Primary infection by the VZV, a DNA alphaherpesvirus, occurs during pregnancy in susceptible women, that is, those without prior exposure or vaccination against the virus. Thus, it presents the potential for complications and adverse outcomes for the pregnant woman and the fetus [2,11].

There are two important distinct clinical manifestations: varicella, corresponding to the initial infection, and herpes zoster, resulting from reactivation of the virus latent in the sensory ganglia. The primary infection presents an incubation period of 10 to 21 days after exposure to the virus. After this interval, the clinical picture begins, including fever, malaise, headache, anorexia, and nonspecific symptoms similar to those of other viral infections. Subsequently, the typical exanthem of the disease appears, composed of macules, papules, vesicles, and crusts, present mainly on the trunk, face, and scalp, associated with intense pruritus [2,11,18].

The physiological changes inherent to pregnancy, especially those related to the immune and respiratory systems, may increase the risk of more severe forms of the disease. Among the important complications associated with morbidity and mortality, varicella pneumonia stands out, in which the main signs and symptoms include dyspnea, cough, chest pain, persistent fever, and hypoxemia, and which may progress to acute respiratory failure and the need for intensive support [4,11,18,19]. Although less frequent, neurological and hepatic complications and secondary bacterial infections of the skin lesions have also been described [2].

In addition to maternal complications, vertical transmission can occur during maternal viremia. Especially when infection occurs between the 8th and the 20th week of gestation, there is a risk of developing CVS, characterized by cutaneous scarring, limb hypoplasia, and neurological and ophthalmological changes. The estimated risk is lower than 2% of cases of maternal infection in this period [20,21]. Infection close to delivery, on the other hand, can cause neonatal varicella, the severity of which depends on the passive transfer of maternal antibodies to the fetus [4]. The most severe cases are especially observed when the maternal exanthem appears between five days before and two days after birth, a period in which there is not enough time for the production and transfer of protective antibodies to the newborn [2,11,19].

### 5.2. Differential Diagnosis

The diagnosis of varicella is predominantly clinical, based on the patient’s signs and symptoms, mainly the typical vesicular exanthem associated with a compatible epidemiological history. However, there are several similar manifestations, both dermatological and infectious, that may present similarities and diagnostic uncertainty, thus requiring an adequate differential diagnosis [2,11].

One of the main diseases confused with varicella is infection by the herpes simplex virus, which produces a picture of grouped and painful vesicular lesions with a localized distribution. Another infection that should be considered as a differential diagnosis is bullous impetigo, caused mainly by *Staphylococcus aureus* [2,4]. Other viral exanthematous diseases, such as measles and rubella, may present with fever and cutaneous manifestations, but generally do not present lesions in multiple evolutionary stages simultaneously, a classic characteristic of varicella that should be considered when making the diagnosis [2,4,11].

In situations of diagnostic uncertainty, where the clinical diagnosis is not sufficient, especially in pregnant women at increased risk of complications, combining the clinical diagnosis with laboratory tests—such as the polymerase chain reaction (PCR) in samples collected from the vesicles—is the most sensitive and specific method of choice. Serological tests are also useful for assessing prior immunity; the detection of IgM and IgG antibodies may complement the investigation in specific situations [2,12,13].

### 5.3. Herpes Zoster During Pregnancy

Herpes zoster is the result of the reactivation of the varicella–zoster virus that was latent in the sensory ganglia after prior infection by varicella. This contrasts with varicella, which represents the primary VZV infection and occurs in pregnant women who have not previously been exposed to the virus or vaccinated against it, and therefore lack protective immunity [4,12].

It presents clinically with neuropathic pain, paresthesias, or a burning sensation, followed by the appearance of grouped vesicles on an erythematous base distributed along a specific dermatome and generally in a unilateral manner [12,13]. The thoracic dermatomes are usually the most affected [15].

The development of herpes zoster during pregnancy is uncommon and has a favorable prognosis. Due to the maternal antibodies against VZV, the risk of transplacental transmission is extremely low and viral reactivation is not associated with CVS, unlike what occurs in primary infection [10,14]. Despite the favorable prognosis, there are some maternal complications, which include post-herpetic neuralgia, secondary bacterial infection of the lesions, and systemic dissemination in immunocompromised patients, although this is rare [15].

Treatment is based on the early use of antivirals, such as acyclovir or valacyclovir, which reduce the duration of symptoms and improve the resolution of the lesions [2,12,13]. Therefore, despite sharing the same etiological agent, primary varicella and herpes zoster present important differences regarding pathophysiology, fetal risk, and gestational prognosis, and it is important to distinguish between them during prenatal follow-up [4,12,15].

## 6. Maternal Complications

Although varicella is a generally benign and self-limited infection in childhood, its occurrence during pregnancy is associated with greater morbidity and mortality compared with non-pregnant women. The physiological and immunological modifications inherent to pregnancy, including changes in cell-mediated immunity, increased oxygen consumption, elevation of the diaphragm, and reduction in the pulmonary functional residual capacity, contribute to a greater susceptibility to severe forms of the disease. Varicella pneumonia represents the most frequent and potentially fatal complication, although neurological and hepatic manifestations and disseminated disease may also occur, particularly in immunosuppressed patients [2,3,4,19].

### 6.1. Varicella Pneumonia

Varicella pneumonia constitutes the main maternal complication associated with primary infection by the VZV, occurring in approximately 10–20% of affected pregnant women [2,4]. Although rare, it is responsible for most of the maternal morbidity and mortality related to the disease and presents a potentially rapid progression to acute respiratory failure.

The pathophysiology of varicella pneumonia is related to the hematogenous dissemination of VZV during the viremia phase, culminating in invasion of the pulmonary parenchyma, an intense local inflammatory response, and diffuse alveolar injury. The physiological changes in pregnancy, such as reduction in the pulmonary functional residual capacity, increased oxygen consumption, and relative reduction in T-cell-mediated immunity, favor a greater severity of the infection [2,3].

The clinical manifestations generally arise between one and six days after the appearance of the cutaneous exanthem and include persistent fever, dry cough, progressive dyspnea, tachypnea, chest pain, and hypoxemia. In severe cases, progression to acute respiratory distress syndrome (ARDS), the need for mechanical ventilation, and intensive support may occur [4].

Several factors are associated with a worse prognosis, including smoking, the presence of more than 100 skin lesions, the third gestational trimester, delay in the initiation of antiviral treatment, and states of immunosuppression [2]. Early recognition of the complication is fundamental, since immediate treatment with intravenous acyclovir is associated with a significant reduction in maternal morbidity and mortality [4,19].

### 6.2. Neurological Complications

The neurological manifestations associated with VZV infection are uncommon but potentially serious. Involvement of the central nervous system can occur through direct viral invasion or through inflammatory and vasculopathic mechanisms induced by the virus [2].

Among the main complications described are encephalitis, aseptic meningitis, cerebellitis, transverse myelitis, and cerebral vasculopathies. Encephalitis represents the most severe form and is characterized clinically by fever, intense headache, alteration of the level of consciousness, seizures, focal neurological deficit, and a decline in mental status [2,22].

The diagnosis is made through analysis of the cerebrospinal fluid associated with detection of viral DNA by PCR, considered the most sensitive and specific method. Magnetic resonance imaging may demonstrate changes compatible with inflammation of the central nervous system [22].

Therapy consists of hospitalization and administration of intravenous acyclovir, since active viral replication constitutes one of the main mechanisms involved in the neurological injury. Early treatment is essential to reduce mortality and permanent neurological sequelae [2,4].

### 6.3. Hepatitis and Disseminated Disease

VZV hepatitis is a rare complication but one of high severity, frequently associated with disseminated forms of the disease. Its occurrence is more frequent in immunocompromised patients, although it may also be observed in previously healthy pregnant women [2].

Systemic dissemination of the virus may result in the simultaneous involvement of multiple organs, including the lungs, liver, central nervous system, and hematological system. Clinically, patients may present with persistent fever, abdominal pain, nausea, vomiting, jaundice, and a marked elevation of aminotransferases [4].

In severe cases, acute hepatic failure, disseminated intravascular coagulation, encephalitis, myocarditis, and multiple organ failure may occur as manifestations of disseminated VZV disease [2,4].

Conditions associated with immunosuppression, such as HIV infection, prolonged use of corticosteroids, chemotherapy, or immunosuppressive therapies, substantially increase the risk of viral dissemination [2]. The early institution of intravenous acyclovir and intensive support measures are fundamental for the improvement of the prognosis [4].

### 6.4. Intensive Care and Maternal Mortality

Severe forms of VZV infection may require admission to an intensive care unit (ICU), especially when there is important respiratory compromise, acute respiratory failure, the need for mechanical ventilation, or multisystem involvement [2].

Historically, before the availability of antiviral therapy, varicella pneumonia presented extremely high maternal mortality rates, reaching values close to 40%. The introduction of intravenous acyclovir, associated with advances in intensive care and ventilatory support, promoted a significant reduction in these indices over the past decades [4,19].

The main factors associated with a worse prognosis include severe hypoxemia, the need for mechanical ventilation, acute respiratory distress syndrome, delay in the initiation of antiviral treatment, and the presence of immunosuppression [2,4]. Although mortality has decreased substantially with therapeutic advances, primary VZV infection continues to be considered a potentially fatal condition during pregnancy, justifying early diagnosis and aggressive management of the complicated forms [2,3,4,19].

## 7. Fetal and Congenital Consequences

Maternal primary infection by the varicella–zoster virus (VZV) during pregnancy may result in transplacental transmission and cause various fetal repercussions, the severity of which is directly related to the gestational period in which the maternal viremia occurs. Although most cases evolve without significant fetal compromise, CVS constitutes the most severe complication of intrauterine infection and is associated with high rates of neonatal morbidity and mortality [2,3,4]. In addition to the classic congenital manifestations, fetal infection may remain latent and manifest later as herpes zoster in childhood [2].

### 7.1. Congenital Varicella Syndrome

CVS is a rare but potentially devastating condition, resulting from fetal infection after the transplacental transmission of VZV during maternal primary infection. The global incidence is estimated at approximately 0.5% to 2% when infection occurs in the first 20 weeks of gestation, with the greatest risk observed between the 13th and 20th weeks [2,14,20].

**Figure 2:**
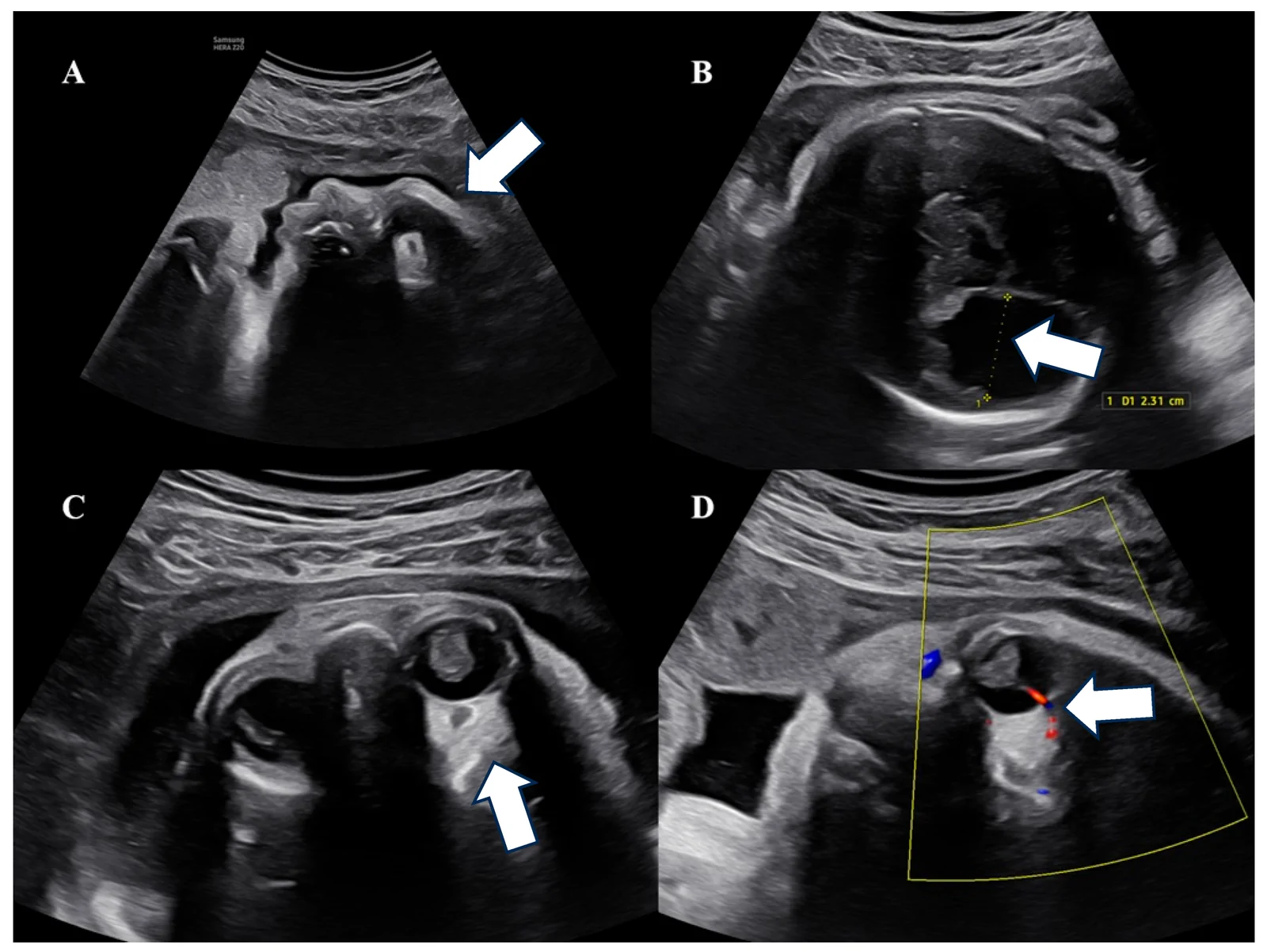
Two-dimensional ultrasound findings of a fetus with congenital varicella syndrome. (**A**) Fetal profile with frontal flattening and microcephaly; (**B**) transventricular view with severe ventriculomegaly; (**C**) axial view of the orbits showing vitreous and lens hyperechogenicity; and (**D**) parasagittal view with the central retinal artery identified via color Doppler. White arrows highlight the characteristic sonographic abnormalities in each image.

The clinical manifestations result from viral tropism for developing tissues and are characterized by a variable spectrum of anomalies. The cutaneous changes are considered classic findings, manifesting in the form of atrophic scars in a dermatomal distribution. Musculoskeletal changes, such as limb hypoplasia and bone deformities, are frequent. Neurological involvement may include microcephaly, cortical atrophy, seizures, neuropsychomotor developmental delay, and cognitive deficit. The most described ophthalmological manifestations are microphthalmia, cataract, chorioretinitis, and optic atrophy (Figure 2). Changes in the gastrointestinal and genitourinary tract may also be present [2,3,4].

Neonatal mortality is significant, possibly reaching approximately 30% in the first months of life. Among survivors, permanent neurological and motor sequelae are frequent, reinforcing the importance of specialized prenatal diagnosis and follow-up [2,14].

### 7.2. Pathogenesis of Fetal Injury

The pathophysiology of fetal injury is related to maternal viremia and the subsequent hematogenous dissemination of VZV through the placenta. After reaching the fetal circulation, the virus presents tropism for ectodermal and neural tissues, promoting a direct cytopathic effect, an inflammatory response, and vascular injury [2,4].

In addition to direct cellular destruction, it is believed that the vasculopathy induced by VZV plays a fundamental role in the genesis of the congenital malformations. Vascular inflammation and the consequent tissue ischemia explain the characteristic segmental distribution of the cutaneous scars and the limb hypoplasia observed in CVS [3,8].

Involvement during the organogenesis phase presents a greater teratogenic potential, since it directly interferes with the normal development of the nervous, ocular, and musculoskeletal systems. Thus, the fetal manifestations result from the combination of viral replication, tissue necrosis, vascular compromise, and inflammatory changes [2].

### 7.3. Timing of Infection and Fetal Risk

The fetal consequences vary substantially according to the gestational age at which maternal infection occurs. When infection occurs before the 13th week, the absolute risk of CVS is low, but the repercussions may be severe due to the critical period of organogenesis [2].

Between the 13th and 20th weeks is the phase of greatest fetal susceptibility, with an approximate risk of 2% being described for the development of CVS [2,14,20].

After the 20th week of gestation, the occurrence of CVS becomes exceptional. However, the virus may remain in a latent state in the fetal sensory ganglia, predisposing to the early appearance of herpes zoster in childhood [2].

When maternal infection occurs in the peripartum period, especially between five days before and two days after delivery, the newborn presents a high risk of developing severe neonatal varicella. In this situation, there is not enough time for the production and transplacental transfer of protective maternal antibodies, which significantly increases neonatal morbidity and mortality [2,4]. Fetal and neonatal risk according to the timing of maternal infection is summarized in Table 3.

### 7.4. Prenatal Diagnosis

Pregnant women affected by primary VZV infection should be referred for follow-up at a service specialized in fetal medicine. Follow-up is based mainly on the performance of serial ultrasound examinations and, in selected situations, on molecular investigation [2,4].

### 7.5. Ultrasound Findings

Serial obstetric ultrasonography constitutes the main tool for fetal monitoring after maternal infection. The changes may arise several weeks after the acute episode, which is why prolonged follow-up is recommended [2,4].

The most frequently described ultrasound findings include intrauterine growth restriction, limb hypoplasia, microcephaly, ventriculomegaly, hydrocephalus, intracranial calcifications, cerebral atrophy, and fetal hydrops [2,3,4,23].

Visceral changes may also be observed, such as intestinal hyperechogenicity, polyhydramnios, bladder dilation, and abnormalities of the urinary tract. Ocular involvement may manifest in the form of microphthalmia and congenital cataract [2,3].

Although the absence of ultrasound changes is associated with a favorable prognosis, it does not completely exclude the possibility of late sequelae. In this way, specialized fetal surveillance is fundamental for parental counseling and for the adequate planning of perinatal care [4,8].

## 8. Neonatal Varicella

### 8.1. Timing of Maternal Infection and Neonatal Risk

The risk of neonatal varicella is directly related to the interval between the onset of cutaneous eruptions in the pregnant woman and delivery. Earlier recommendations commonly used the interval from 5 days before to 2 days after delivery [21]. Current UK guidance considers neonates at highest risk when maternal varicella develops from 7 days before to 7 days after delivery [19]. In this period, there is not enough time for the placental transfer of protective maternal antibodies (IgG) to the fetus, exposing the newborn to a high viral load without passive immunity [10,19]. If maternal infection occurs 1 to 4 weeks before delivery, up to 50% of neonates may be infected, although the disease is generally milder due to the presence of maternal antibodies [10].

### 8.2. Clinical Manifestations

Neonatal varicella acquired in the peripartum period generally manifests between the 5th and the 10th day of life. Newborns may present with a generalized vesicular exanthem, fever, lethargy, and respiratory difficulty. The severe form is characterized by necrotizing varicella pneumonia, hepatitis, encephalitis, and disseminated intravascular coagulation [10,11]. In contrast, infection acquired more than 7 days before delivery is usually milder, with few skin lesions and a benign course [19].

### 8.3. Neonatal Morbidity and Mortality

Before the availability of specific immunoglobulin and antiviral therapy, neonatal mortality from peripartum varicella reached 30% [10]. With the use of immunoglobulin and acyclovir, this rate decreased to approximately 7% [19,24]. Even so, neonatal varicella remains a pediatric emergency, especially in premature infants and very-low-birth-weight newborns [11].

### 8.4. Management of Neonates Exposed to Maternal Varicella

According to current UKHSA/RCOG guidance, neonates born to mothers who develop varicella from 7 days before to 7 days after delivery are considered at highest risk and should receive post-exposure prophylaxis as soon as possible after birth, using VZIG or, when unavailable, IVIG according to local recommendations and product availability [19,25]. Breastfeeding is permitted, provided there are no active lesions on the nipples; in this case, the milk should be expressed and offered to the baby [19]. Current recommendations for neonatal post-exposure prophylaxis and treatment are summarized in Table 4.

## 9. Diagnostic Approach

### 9.1. Clinical Diagnosis

The diagnosis of infection by the Varicella–Zoster virus (VZV) during pregnancy is frequently made on the basis of the characteristic clinical findings, with asymptomatic cases being rare [8]. Varicella typically manifests with a prodrome of fever, malaise, and a pruritic vesicular exanthem, with lesions in different evolutionary stages distributed predominantly on the scalp, face, and trunk [8,11].

In previously susceptible pregnant women presenting with characteristic signs and symptoms, the isolated clinical diagnosis can generally be used. However, situations that present with atypical presentations, an uncertain vaccination history, immunosuppression, or exposure without the development of lesions may require laboratory confirmation [8,15].

Herpes zoster, in turn, is characterized by a painful vesicular eruption limited to one or more dermatomes, generally unilateral, resulting from the reactivation of the VZV latent in the sensory ganglia [4]. The distinction between primary infection and viral reactivation is important due to the greater risk of vertical transmission associated with maternal varicella [15].

### 9.2. Serological Tests

Serology constitutes an important diagnostic tool and one for assessing maternal immunity; however, it is not the most useful method for assessing active disease. Its main role is in pre- or post-vaccination situations, thus being useful for determining whether the pregnant woman is susceptible or immune to infection [4]. Screening for immunity to VZV is recommended even before conception; thus, women who present a negative result for antibodies should receive the vaccine before becoming pregnant [2].

A positive IgM result occurs in primary infection; however, its isolated assessment should not be applied. The positivity of the test may persist for months, thus making assessment difficult in cases of a possible reinfection. IgG, in turn, can be detected early, demonstrating immunity following vaccination or prior contact with the virus, and is associated with a low risk of primary infection after exposure [4]. However, it is necessary to emphasize the limitations present in the assessment of serology, especially with respect to false-positive or false-negative results [2,4].

Although VZV IgM testing may support the diagnosis of primary infection, its interpretation has important limitations. False-positive results may occur because of cross-reactivity with other alphaherpesviruses, particularly herpes simplex virus type 1 and herpes simplex virus type 2, while false-negative results may be observed early in the course of infection or in immunocompromised individuals. Therefore, IgM results should always be interpreted in conjunction with the clinical presentation and, whenever possible, confirmed by molecular methods such as PCR. In cases where the timing of maternal infection remains uncertain, VZV IgG avidity testing may provide additional diagnostic value. High-avidity IgG antibodies generally indicate past infection, whereas low-avidity antibodies are consistent with recent primary infection, thereby helping to estimate the timing of infection and supporting clinical decisions regarding fetal surveillance and neonatal management.

### 9.3. Polymerase Chain Reaction

The PCR test is considered the most sensitive and specific laboratory method for detection of the viral DNA [4]. The test can be performed on samples of vesicular fluid, blood, or amniotic fluid, making diagnostic confirmation possible in different presentations, including those with atypical clinical features [8,15].

In addition, the PCR is the main test for demonstrating fetal infection through amniocentesis [11]. PCR testing should be performed after an adequate interval between maternal infection and sample collection to maintain its high sensitivity for detecting viral DNA [8,15].

The identification of VZV in the amniotic fluid confirms fetal infection; however, its result should be analyzed together with repeated ultrasound examinations, in order to assess the development of CVS and/or signs of severity in fetal involvement [8,11,15].

### 9.4. Prenatal Assessment After Maternal Infection

Transmission of the varicella virus can occur in three different periods: prenatal, perinatal, or postnatal. In the prenatal period, transmission occurs mainly through the transplacental route, as a consequence of maternal viremia [4].

Pregnant women with primary infection by the virus should be referred to specialists in fetal medicine, especially between the 16th and 20th weeks of gestation or within the first five weeks after diagnosis of the infection. In this period, the main method of fetal assessment is serial ultrasonography, performed in order to assess findings such as fetal growth restriction, limb hypoplasia, ventriculomegaly, hydrocephalus, polyhydramnios, among other manifestations characteristic of CVS [4,8,11].

The risk of fetal anomalies is lower when maternal infection occurs later, between the 21st and 36th weeks of gestation, presenting favorable prognoses and minimizing fetal consequences. However, this situation does not exclude the need for the repeated ultrasound examinations previously mentioned [2].

## 10. Management of Varicella Exposure During Pregnancy

### 10.1. Assessment of Maternal Susceptibility

The first step when facing a contact is to determine the immunological status of the pregnant woman. Women with an unequivocal clinical history of varicella or with proof of complete vaccination (two doses) are considered immune [19,25]. For those without a history or with a doubtful history, serology for anti-VZV IgG should be requested. A positive result within 10 days after the contact indicates prior immunity [4].

### 10.2. Definition of Significant Exposure

According to the current UKHSA and RCOG guidance, significant exposure includes face-to-face contact with an infectious individual, being in the same room for at least 15 min, or contact in a large open ward [19,25]. Because definitions vary across jurisdictions, clinicians should refer to the recommendations applicable in their own setting. Hospitalized patients who share the same room with a case are also considered exposed [13].

### 10.3. Post-Exposure Prophylaxis

Recent recommendations from the UK Health Security Agency (UKHSA), subsequently incorporated into the 2024 Royal College of Obstetricians and Gynaecologists (RCOG) Green-top Guideline, recommend oral antiviral therapy as the preferred first-line post-exposure prophylaxis for susceptible immunocompetent pregnant women. However, this recommendation is not universal, and other organizations, including the Society of Obstetricians and Gynaecologists of Canada (SOGC), continue to recommend VZIG as first-line prophylaxis.

Susceptible (non-immune) pregnant women who have had a significant exposure to the VZV should receive post-exposure prophylaxis (PEP) to reduce the risk of maternal infection and its complications. Traditionally, specific immunoglobulin against VZV (VZIG) was considered the main prophylactic strategy, and should be administered as early as possible, ideally within the first 96 h after exposure, and may still be used up to 10 days after the contact [10,11,19,24].

However, more recent recommendations from the UK Health Security Agency [26] have come to indicate oral antiviral therapy as the first choice for post-exposure prophylaxis in susceptible immunocompetent pregnant women. The recommended regimen consists of acyclovir 800 mg orally, four times a day, or valacyclovir 1000 mg orally, three times a day, administered between the 7th and the 14th day after exposure to VZV [19]. The delayed initiation of antiviral therapy is based on evidence that demonstrated greater efficacy in the prevention of clinical varicella when treatment is started seven days after exposure, compared with immediate use [19].

Acyclovir remains the preferential option during pregnancy due to the greater volume of available data on its maternal–fetal safety. Valacyclovir, with greater bioavailability and a simpler dosing regimen, may be considered when there is concern about treatment adherence [19].

VZIG remains an alternative in cases in which antivirals are contraindicated or not tolerated. In this situation, its administration should occur as early as possible and, at the latest, up to 10 days after exposure to the virus [19].

### 10.4. Varicella–Zoster Immunoglobulin (VZIG)

VZIG is a preparation of human IgG with high titers of anti-VZV antibodies, used as post-exposure prophylaxis in susceptible pregnant women when antivirals are contraindicated or not tolerated [11,25].

The dose recommended in the United States is 125 U/10 kg of body weight, up to a maximum of 625 U, by the intramuscular route. Alternatively, it can be administered by the intravenous route at a dose of 1 mg/kg, which provides protective serum levels more rapidly [11,13]. Adverse effects are uncommon and generally mild, including pain at the application site and headache [18].

The protection conferred by VZIG lasts approximately three weeks, and a new administration may be necessary in the event of re-exposure in this period [11,24]. In addition to reducing the incidence of maternal varicella by about 50%, VZIG may also attenuate the severity of the disease when infection occurs despite prophylaxis [24]. It should also be considered that its use may prolong the incubation period, justifying more prolonged clinical follow-up after exposure [11].

### 10.5. Antiviral Prophylaxis

Vaccination is the main strategy for the prevention of infection by the VZV in susceptible women of reproductive age. The vaccine contains live attenuated virus and has demonstrated a significant reduction in the incidence and mortality related to varicella [19].

Immunization is recommended for women without evidence of immunity before pregnancy or in the postpartum period. The regimen consists of two doses with an interval of 4 to 8 weeks, with the recommendation to avoid pregnancy for at least 1 month after vaccination [13,19,27].

As this is a live attenuated virus vaccine, its use is contraindicated during pregnancy. However, cases of inadvertent vaccination have not been associated with an increased risk of CVS or fetal malformations [13,19]. Susceptible pregnant women identified during prenatal care should be advised to receive the vaccine after delivery, with immunization being considered safe during breastfeeding [19].

## 11. Treatment of Maternal Varicella Infection

### 11.1. Outpatient Management

Most pregnant women with uncomplicated varicella can be managed on an outpatient basis. Isolation should be advised (avoiding contact with other pregnant women and newborns) until all lesions have crusted over (about 5 days from the onset of the appearance of the lesions) [11,19]. Symptomatic measures include antipyretics, antihistamines for pruritus, and local hygiene to prevent secondary bacterial infection [10,11].

### 11.2. Oral Antiviral Therapy

Oral acyclovir (800 mg five times daily for 7 days) or valacyclovir (1 g three times daily for 7 days) should be initiated as early as possible, ideally within the first 24 h after rash onset. Current UK guidelines (RCOG/UKHSA) recommend oral acyclovir for pregnant women presenting within 24 h of rash onset at ≥20 weeks’ gestation, whereas before 20 weeks, treatment should be considered following an individualized discussion of the potential benefits and uncertainties. Recommendations from other national guidelines differ regarding antiviral treatment in early pregnancy [4,13,19]. Studies show that early treatment reduces the duration of fever and the severity of maternal symptoms [18]. Although there is no conclusive evidence that it prevents CVS, acyclovir crosses the placenta and may reduce maternal viremia [11,13]. Large registries of pregnant women exposed to acyclovir have not demonstrated an increased risk of congenital malformations, including when used in the first trimester [18].

### 11.3. Indications for Hospitalization

Hospital admission is indicated in the following situations: the presence of respiratory symptoms (cough, dyspnea, chest pain); neurological signs (intense headache, photophobia, seizures); a hemorrhagic or very dense rash (more than 100 lesions); persistent fever or the appearance of new lesions after the 6th day of disease; immunosuppression (including the use of corticosteroids); and proximity to term (≥36 weeks) [2,5,6]. The patient should preferably be admitted to a non-obstetric unit (an infectious diseases ward or isolation), to avoid contagion of other vulnerable pregnant women [28].

### 11.4. Intravenous Acyclovir

In complicated varicella, especially pneumonia, intravenous acyclovir should be administered at a dose of 10 to 15 mg/kg every 8 h for 5 to 10 days [10,13,18]. Intravenous therapy is also indicated for cases of encephalitis, severe hepatitis, or hemorrhagic varicella [19]. Historically, mortality from varicella pneumonia in pregnancy reached 20–45% in the pre-antiviral era; with intravenous acyclovir and intensive support, current mortality is about 3–14% [10,19]. Maternal antiviral therapy and post-exposure prophylaxis according to clinical scenario are summarized in Table 5.

### 11.5. Supportive Care and Intensive Care

Pregnant women with varicella pneumonia may progress rapidly to hypoxemic respiratory failure. Up to 40% of these patients require mechanical ventilation [11,18]. Management should be multidisciplinary, involving an obstetrician, intensivist, infectious disease specialist, and neonatologist. The decision to expedite delivery in pregnant women with respiratory failure is individualized, aiming to improve respiratory mechanics and facilitate maternal care [19]. In term pregnancies, when maternal varicella infection occurs close to delivery, elective postponement of delivery for at least 5 to 7 days after the onset of the exanthem may be considered, whenever maternal and fetal clinical conditions allow, with the objective of favoring the placental transfer of protective IgG antibodies to the fetus and thus reducing the risk of severe neonatal varicella [19,25]. This management, although based on solid pathophysiological foundations, is based on limited clinical experience [19].

## 12. Obstetric Management

### 12.1. Fetal Surveillance

Fetal surveillance represents a fundamental component of the obstetric management of infected patients, especially when infection occurs before 20 weeks of gestation. It is essential that fetal assessment be initiated approximately five weeks after maternal infection or at an interval sufficient for possible fetal changes to be perceived [2,15,29].

Serially performed ultrasonography is the main method for the assessment and monitoring of possible fetal changes after maternal viral detection. It is recommended that the ultrasound examination be performed in detail, directed at identifying possible fetal changes compatible with infection [2,15,29].

### 12.2. Timing of Delivery

The timing of delivery should be assessed in an individualized manner, taking into consideration decisions based on maternal condition, gestational age, and the period of onset of the infection, always paying close attention to ensuring that maternal–fetal safety is not compromised [2,15,19].

The main obstetric concern involves the period in which the mother developed the infection. Thus, greater attention is required for infections established in the period between five days before and two days after delivery, a situation that poses a greater risk of severe neonatal varicella due to the inadequate transmission of protective maternal antibodies to the fetus [2,19].

In cases in which the maternal and fetal clinical conditions are favorable, postponement of delivery may be chosen, seeking a period sufficient for the transplacental transfer of antibodies to be satisfactory, thus reducing the risk of severe neonatal disease [2,15,19].

### 12.3. Delivery Planning in Active Maternal Infection

Obstetric planning in the presence of active maternal infection close to term should be carried out in the usual manner, with a complete multidisciplinary assessment focused simultaneously on the maternal clinical status and fetal well-being always being necessary [2,15].

There is no evidence to support a specific mode of delivery to be followed; this should be guided by the usual obstetric indications. The performance of a cesarean section should be based solely on the maternal–fetal assessment, with its routine practice not being necessary exclusively on account of the viral infection in question [2,15,19].

Pregnant women who present the more severe forms of the disease, especially those associated with pneumonia caused by the varicella–zoster virus, require intensive monitoring and adequate treatment throughout the period of the disease, maintaining such management at the time of delivery [2,19].

Neonatal assessment should be performed in a multidisciplinary manner, especially in cases of high-risk exposure resulting from peripartum maternal infection. The definition of prophylactic and therapeutic measures should be carried out jointly with the neonatology team, in order to establish the care necessary for the newborn [2,15,19].

### 12.4. Isolation Precautions

The main routes of transmission of the virus are through the respiratory route and through direct contact with the secretion originating from the cutaneous lesions. Infected pregnant women require respiratory and contact precautions until all lesions have evolved to the crust stage; in these cases, adequate covering of the varicella exanthem is recommended [2,15,19]. In addition, attention is necessary to the care aimed at preventing transmission to individuals susceptible to infection, such as: health professionals, newborns, and other pregnant women [2,15].

### 12.5. Considerations Regarding Breastfeeding

Breastfeeding is, in the great majority of cases, maintained normally, and its suspension in the presence of the infection is not necessary. However, it is only possible to carry it out when there are no active lesions in the region of the breast and areola, in addition to rigorous precautions for the prevention of postnatal transmission, including adequate hand hygiene and appropriate covering of lesions located in other regions of the body [2,3].

In cases of lesions in regions of the nipple and/or areola, direct breastfeeding on the affected breast should be avoided until the complete resolution of the lesions, that is, until they turn into crusts. Thus, expression of breast milk may be carried out so that lactation is maintained until resolution of the maternal condition. Appropriate neonatal assessments and guidance regarding infection control and adequate hygiene practices are necessary [2,3].

## 13. Vaccination and Prevention Strategies

### 13.1. Preconception Vaccination

The prevention of varicella in pregnancy constitutes one of the fundamental pillars in the reduction in the maternal–fetal morbidity and mortality associated with VZV, a highly contagious herpesvirus responsible for primary systemic infection and the potential establishment of latency with subsequent reactivation [11]. Transmission occurs predominantly through the respiratory route and direct contact with cutaneous lesions, presenting a high attack rate in susceptible individuals [16].

Although varicella is frequently benign in childhood, its occurrence in adults, especially pregnant women, is associated with a greater risk of severe complications, such as pneumonia, encephalitis, and vertical transmission with adverse fetal outcomes [12,21]. Infection in pregnancy, although relatively rare, remains relevant from a clinical and epidemiological point of view, with an estimated incidence between 0.5 and 3 cases per 1000 pregnancies [12,16].

In this context, preconception vaccination represents the most effective strategy of primary prevention. The vaccines against VZV, composed of live attenuated virus, induce a robust humoral and cellular immune response and confer lasting protection against primary infection [5]. International guidelines recommend that women of reproductive age without evidence of immunity be vaccinated before conception, with a minimum interval of four weeks between vaccination and pregnancy [18,19].

The identification of susceptibility should be carried out through a reliable clinical history or serology for anti-VZV IgG in doubtful cases [18,19]. The systematic implementation of this strategy significantly reduces the incidence of maternal varicella and of CVS, a rare but serious condition associated with multiple fetal malformations [21,30].

### 13.2. Postpartum Vaccination

Postpartum vaccination constitutes an essential strategic intervention for women identified as susceptible during prenatal care. Unlike during pregnancy, the vaccine is considered safe in the puerperium, including during breastfeeding, with no evidence of transmission of the vaccine virus through breast milk [18,19].

It is recommended that the first dose be administered during the postpartum hospitalization itself or at the first puerperal consultation, with subsequent scheduling of the second dose according to the vaccination schedule [18,19]. This approach reduces the risk of infection in future pregnancies and contributes to the reduction in viral circulation in the community.

### 13.3. Inadvertent Vaccination During Pregnancy

Although the vaccine against varicella is formally contraindicated during pregnancy because it is a live attenuated virus [19], situations of inadvertent administration may occur.

Evidence from surveillance registries and observational studies demonstrates that there is no association between inadvertent vaccination and an increased risk of miscarriage, congenital malformations, or CVS [6,7]. Thus, accidental exposure does not constitute an indication for termination of pregnancy, with only adequate counseling and routine prenatal follow-up being recommended [18,31].

### 13.4. Public Health Implications

The implications of varicella in pregnancy transcend individual care and assume relevance in public health. Maternal infection may progress to severe forms, especially varicella pneumonia, associated with greater morbidity and mortality [12,21]. In addition, vertical transmission may result in CVS or severe neonatal varicella [21,30].

The introduction of universal vaccination programs has resulted in a substantial reduction in the incidence of the disease, hospitalizations, and VZV-related deaths [16]. The decrease in viral circulation contributes directly to the indirect protection of vulnerable groups, including pregnant women and newborns.

### 13.5. Herd Immunity and Global Prevention Strategies

Herd immunity plays a central role in the prevention of varicella in pregnancy. In populations with high vaccination coverage, a significant reduction in the circulation of VZV is observed, decreasing the probability of exposure of susceptible individuals [16].

Effective global strategies include universal childhood vaccination, the screening of susceptibility in women of childbearing age, educational campaigns aimed at reproductive planning, and standardized protocols for post-exposure prophylaxis during pregnancy [19,29].

The integration between primary care, reproductive health, and prenatal care is fundamental for the early identification of susceptible women and the implementation of preventive measures. Thus, the prevention of varicella in pregnancy should be understood as a care continuum, initiated in the preconception period, reinforced during prenatal care, and consolidated in the puerperium [11,18,19].

## 14. International Guidelines and Differences in Clinical Practice

The recommendations of the United Kingdom Health Security Agency (UKHSA), the Royal College of Obstetricians and Gynaecologists (RCOG), the Society of Obstetricians and Gynaecologists of Canada (SOGC), and National Health Service (NHS) protocols differ in several aspects, particularly regarding post-exposure prophylaxis and clinical management, although they share broad agreement on the overall approach to maternal assessment and treatment [18,19,25,26]. However, there is a consensus among the associations on the potential for morbidity and severity of the disease in pregnant women and the need for a specific clinical approach [18,19,25,26]. The assessment of maternal immunity represents a relevant topic and one of greater consensus among the guidelines: the need to identify women susceptible to VZV during prenatal follow-up.

To facilitate comparison, the principal recommendations and areas of agreement and divergence among the current international guidelines are summarized in Table 6.

The RCOG recommends that all pregnant women be questioned about a history of varicella, herpes zoster, and vaccination regarding varicella at the first prenatal consultation [19]. The SOGC, in addition to the investigation, recommends serological confirmation in the case of diagnostic uncertainty [18], evidencing a greater level of detail of screening in the Canadian context. The NHS adopts similar guidance, emphasizing that women known to be seronegative should avoid contact with infected individuals [25].

The most relevant divergences between the current guidelines are observed in post-exposure prophylaxis. Over the years, VZIG constituted the main preventive strategy for susceptible pregnant women who were exposed to the virus. However, following updates made by the United Kingdom Health Security Agency [26], incorporated by the RCOG in 2024, oral acyclovir has come to be recommended as the first choice for post-exposure prophylaxis, to be initiated between the seventh and the fourteenth day after exposure to the virus [19]. Currently, VZIG is being reserved for situations in which there are contraindications to the use of antivirals or specific clinical circumstances [19,25]. In contrast, the Canadian SOGC guideline, published before these updates, maintains VZIG as the main prophylactic strategy [18].

Regarding the treatment of established maternal infection, there is international consensus regarding the benefit of early antiviral treatment, mainly when initiated within the first 24 h after the appearance of the cutaneous lesions [18,19,25]. Treatment is associated with a reduction in the duration of symptoms and in the severity of the disease. In severe cases, especially in the presence of respiratory compromise, all guidelines recommend hospitalization and intravenous administration of acyclovir [18,19,25].

Another area of agreement refers to fetal surveillance after maternal infection, with specialized fetal follow-up and ultrasound follow-up being of extreme importance for the assessment of possible signs of CVS, such as fetal growth restriction, limb hypoplasia, intracranial calcifications, microcephaly, and others [18,21].

There is consensus among the RCOG, SOGC, and other international guidelines that the vaccine against varicella, composed of live attenuated virus, is contraindicated during pregnancy [18,19]. However, all recommend preconception vaccination for susceptible women identified before pregnancy. The guidelines also agree that seronegative women identified during pregnancy should receive vaccination in the postpartum period, including during breastfeeding, since there is no evidence of significant risk to the infant [18,19].

**Table 6:** Comparison of Current International Guidelines for the Management of Varicella Exposure During Pregnancy.

Guideline	Country	Year	Significant Exposure	Immunity Assessment	First-Line PEP	Timing	Maternal Treatment	Neonatal Prophylaxis	Evidence/Comments
UKHSA	UK	2025	Household exposure, face-to-face contact, or contact in the same room for ≥15 min during the infectious period	History of varicella/vaccination; VZV IgG if uncertain	Oral acyclovir/valacyclovir	Days 7–14	Oral/IV acyclovir	Risk-based: IV varicella immunoglobulin/IVIG plus aciclovir for Group 1 neonates; oral antivirals for other eligible high-risk infants	Updated national guidance
NHS Lothian	UK	2025	As defined by UKHSA	History and VZV IgG if required	Oral antivirals	Days 7–14	Oral/IV acyclovir	Refer to UKHSA neonatal PEP guidance	Local implementation of UK policy
RCOG	UK	2024	Contact in the same room for ≥15 min, face-to-face contact, or relevant continuous/multiple exposure during the infectious period	History and VZV IgG if required	Oral antivirals	Days 7–14	Oral/IV acyclovir	Outside the scope of the guideline; refer to UKHSA guidance	Incorporates UKHSA recommendations
SOGC	Canada	2012	Significant close exposure to infectious varicella	History and serology if uncertain	VZIG	Within 96 h	Oral acyclovir for clinically significant infection; IV acyclovir for severe disease	VZIG for eligible exposed neonates	Older guideline; predates current UK antiviral-first PEP recommendations

Abbreviations: ACV, acyclovir; IV, intravenous; NHS, National Health Service; PEP, post-exposure prophylaxis; RCOG, Royal College of Obstetricians and Gynaecologists; SOGC, Society of Obstetricians and Gynaecologists of Canada; UK, United Kingdom; UKHSA, UK Health Security Agency; NHS Lothian (National Health Service Lothian); VZIG, varicella–zoster immunoglobulin; VZV, varicella–zoster virus; IgG, immunoglobulin G.

## 15. Future Perspectives and Research Gaps

In recent years, the incidence of varicella has decreased significantly, especially in countries with high vaccination coverage rates. Despite this reduction, the infection continues to be a relevant concern during pregnancy due to the potential for maternal, fetal, and neonatal complications. Although the advances achieved over the past decades have contributed to the improvement of the management of infection by the VZV, important knowledge gaps still persist that limit the formulation of clinical recommendations grounded in robust evidence. As a consequence, a large part of the international guidelines is based predominantly on observational studies, case series, and expert consensus, rather than on prospective clinical trials of high methodological quality [11,19].

One of the main gaps identified in the literature refers to the scarcity of prospective clinical trials that assess the comparative efficacy between VZIG and the antivirals used in post-exposure prophylaxis. In recent years, some guidelines have come to prioritize the use of antivirals, especially acyclovir, over the routine use of VZIG. However, this change has been supported mainly by indirect evidence and observational studies, since the data from high-quality comparative studies are still limited. Thus, the definition of the most effective prophylactic strategy for the prevention of infection and its associated complications remains uncertain [18,19].

Another relevant area of investigation refers to the safety of the use of antivirals during pregnancy. Although acyclovir is widely recommended in clinical practice and presents a favorable safety profile, there are still limitations related to the availability of prospective studies aimed at the assessment of long-term maternal, fetal, and neonatal outcomes. In this context, multicenter studies with prolonged follow-up could provide more robust evidence regarding possible late effects resulting from fetal exposure to antivirals, contributing to a better understanding of the safety of these therapies during pregnancy [11].

Another important front of research refers to the identification and validation of maternal, fetal, and virological prognostic markers capable of predicting the risk of complications associated with VZV infection during pregnancy. The development of these tools may contribute to a more precise risk stratification, favoring the individualization of prenatal follow-up and the implementation of targeted monitoring strategies. In addition, their application in clinical practice has the potential to reduce diagnostic uncertainties, improve decision-making, and optimize the management of pregnant women exposed to or infected by VZV [11,20,21].

In summary, although significant advances have been achieved in the prevention and management of varicella during pregnancy, challenges still persist related to the quality of the available evidence, the assessment of new prophylactic strategies, and the understanding of the mechanisms involved in the vertical transmission of the virus. The production of more robust evidence may contribute substantially to the improvement of maternal–fetal care and to the reduction in the maternal, fetal, and neonatal morbidity and mortality associated with infection by the varicella–zoster virus.

## 16. Conclusions

VZV infection during pregnancy remains an important clinical concern because of its potential to cause severe maternal, fetal, and neonatal morbidity despite its low incidence. Optimal care relies on the timely identification of susceptible women, accurate diagnosis, appropriate risk stratification following exposure, and prompt implementation of evidence-based therapeutic and preventive measures. As the clinical consequences vary according to the timing of maternal infection, management requires a multidisciplinary and individualized approach throughout pregnancy and the perinatal period.

Although substantial advances have been achieved in antiviral therapy, immunoprophylaxis, and vaccination strategies, uncertainties remain regarding the optimal use of preventive interventions during pregnancy and the long-term safety of antiviral therapy. Continued research, together with improved implementation of preventive strategies and equitable access to care, will be essential to further reduce the burden of VZV-related maternal, fetal, and neonatal disease.

## Figures and Tables

**Table 1:** Comparison of aspects of varicella–zoster infection between temperate and tropical/subtropical regions.

Aspect	Temperate Regions	Tropical/Subtropical Regions
Predominant age of infection	Childhood	Adolescence and adulthood
Seroprevalence in adults	Generally > 90%	Variable (≈25–85%)
Proportion of susceptible adults	Low	Higher
Incidence of primary VZV infection during pregnancy	Approximately 1–5 cases per 10,000 pregnancies	Likely higher in regions with lower seroprevalence, although precise estimates vary
Incidence of herpes zoster during pregnancy	Rare (approximately 1–3 cases per 1000 pregnancies)	Similarly rare; no evidence of increased incidence during pregnancy
Susceptibility among pregnant women	Lower	Higher
Seasonality	More evident (winter/spring)	Less defined
Epidemiological profile	Early infection and broad natural immunization	Late infection and greater immunological heterogeneity
Potential obstetric impact	Lower proportion of susceptible pregnant women	Greater risk of primary infection in pregnancy

**Table 2:** Risk factors associated with maternal varicella–zoster virus infection during pregnancy.

Factor	Reported Association
Smoking	Greater risk of pneumonia [13]
>100 skin lesions	Greater clinical severity [13]
Immunosuppression	Greater risk of viral dissemination [2]
Third trimester	Greater pulmonary severity [13]
Absence of prior immunity	Greater risk of primary infection [4]

Source: Adapted from the literature [2,4,13].

**Table 3:** Fetal and neonatal risk according to the timing of maternal varicella–zoster virus infection.

Timing of Maternal Infection	Principal Embryonic, Fetal, or Neonatal Risk	Approximate Magnitude/Remarks
<13 weeks	Congenital varicella syndrome during organogenesis	Low absolute risk, but potentially severe repercussions [2]
13–20 weeks	Congenital varicella syndrome (period of greatest susceptibility)	Risk ≈ 2% [2,14,20]
>20 weeks	Congenital syndrome exceptional; latent infection	Predisposition to early childhood herpes zoster [2]
Peripartum (current UK guidance: 7 days before to 7 days after delivery; earlier guidelines: 5 days before to 2 days after delivery)	Severe neonatal varicella	Insufficient transfer of maternal antibodies; high neonatal morbidity/mortality (historically ≈ 30%) [2,4,10]
1–4 weeks before delivery	Neonatal varicella (generally milder)	Up to 50% of neonates infected, attenuated by maternal antibodies [10]

Source: the authors, synthesized from the text (Section 5, Section 7 and Section 8).

**Table 4:** Current recommendations for the prevention and treatment of neonatal varicella.

Clinical Scenario	Recommended Management	Timing	Comments/Guideline Considerations
Post-exposure prophylaxis			
Maternal varicella from 7 days before to 7 days after delivery	VZIG (or IVIG if VZIG is unavailable, according to local guidance)	As soon as possible after birth/exposure	Highest-risk neonatal group; recommendations vary according to product availability and national guideline
Premature neonates with significant exposure *	VZIG according to product labeling	As soon as possible after exposure	Criteria for prophylaxis vary among guidelines
Treatment of established disease			
Suspected or confirmed neonatal varicella	IV acyclovir 10–15 mg/kg every 8 h	Immediately after diagnosis or symptom onset	Usually for 5–7 days; longer in severe disease

Abbreviations: IV, intravenous; IVIG, intravenous immunoglobulin; VZIG, varicella–zoster immunoglobulin. * This table summarizes current UKHSA/RCOG recommendations. Eligibility for neonatal post-exposure prophylaxis and the choice between VZIG and IVIG may vary in other national guidelines and according to product availability. Dosing should follow the prescribing information for the specific immunoglobulin preparation used.

**Table 5:** Antiviral therapy and post-exposure prophylaxis for varicella–zoster virus infection during pregnancy according to clinical scenario.

Clinical Scenario	Recommended Agent and Regimen	Timing/Remarks
Post-exposure prophylaxis in susceptible pregnant women without immunocompromising conditions	Oral acyclovir 800 mg four times daily or valacyclovir 1 g three times daily	Days 7–14 after exposure (UKHSA/RCOG first-line recommendation) [19]
Post-exposure prophylaxis when antivirals are contraindicated or not tolerated	VariZIG 125 IU per 10 kg body weight IM (maximum 625 IU) or IVIG 1 mL/kg [0.5 g/kg] IV	As early as possible, up to 10 days after exposure [11,13,19]
Established uncomplicated maternal varicella (outpatient)	Oral acyclovir 800 mg five times daily for 7 days or valacyclovir 1 g three times daily for 7 days	Ideally within 24 h of exanthem onset [4,13,19]
Complicated maternal varicella (pneumonia, encephalitis, severe hepatitis, or hemorrhagic disease)	Intravenous acyclovir 10–15 mg/kg per dose every 8 h for 5–10 days	Hospitalization with multidisciplinary supportive care [10,13,18,19]
Preconception/postpartum prevention	Live attenuated varicella vaccine, 2 doses (4–8-week interval)	Contraindicated during pregnancy; recommended postpartum, including during breastfeeding [13,19]

Source: adapted from the cited references. Abbreviations: IM, intramuscular; IV, intravenous; RCOG, Royal College of Obstetricians and Gynaecologists; UKHSA, United Kingdom Health Security Agency; VZIG, varicella–zoster immunoglobulin; VariZIG, Varicella-Zoster Immune Globulin (Human).

## Data Availability

No new data were created or analyzed in this study. Data sharing is not applicable to this article.
